# RNA methylation in autoimmune rheumatic diseases: mechanisms and therapeutic potential

**DOI:** 10.3389/fimmu.2026.1779307

**Published:** 2026-05-01

**Authors:** Yuyao Wang, Guanhui Song, Yujin Xue, Xiaoya Li, Shiping Cheng, Meijie Liu, Hong Liu, Jinghua Pan, Hongyan Zhao, Bin Liu, Danping Fan

**Affiliations:** 1 Experimental Research Center, China Academy of Chinese Medical Sciences, Beijing, China; 2 The First Clinical Medical School, Shaanxi University of Chinese Medicine, Xianyang, Shaanxi, China; 3 The Institute of Medicinal Plant Development, Chinese Academy of Medical Sciences/Peking Union Medical College, Beijing, China; 4 Clinical Medical College, Jiangxi University of Chinese Medicine, Nanchang, China; 5 Institute of Basic Research in Clinical Medicine, China Academy of Chinese Medical Sciences, Beijing, China

**Keywords:** ankylosing spondylitis, autoimmune rheumatic diseases, primary Sjögren’s syndrome, rheumatoid arthritis, RNA methylation, systemic lupus erythematosus

## Abstract

Autoimmune rheumatic diseases (ARDs), often characterized by pain, constitute a diverse group of autoimmune conditions involving inflammation-mediated injuries to bones, joints, surrounding connective tissues, and occasionally other organs. RNA methylation is a key epitranscriptomic modification that regulates gene expression by influencing stability, splicing, nuclear translocation and degradation. Recent studies have highlighted the crucial role of RNA modification in the pathogenesis and progression of various ARDs. RNA modification affects critical biologic processes of ARDs, such as inflammation, immune response. This review systematically explores the landscape of RNA modification in ARDs, elucidating its regulatory roles and therapeutic implications, including rheumatoid arthritis, systemic lupus erythematosus, ankylosing spondylitis, primary Sjögren’s syndrome, systemic sclerosis. The intricate mechanisms of RNA modification can lead to the development of novel diagnostic biomarkers and therapeutic strategies, ultimately improving patient outcomes.

## 1 Introduction

With the advent of specific, quantitative, precise, and sensitive technologies in RNA modification research, the field of RNA epitranscriptomics has been rapidly expanding since its emergence in 2010 (1, 2). In vertebrates, RNA modifications, acting as dynamic and reversible regulatory layers, have been identified as crucial mechanisms fine-tuning RNA splicing, stability, translation, and localization, thereby profoundly influencing RNA fate and function. Among the plethora of known modifications, methylations constitute a major fraction, methylations modifications occur across diverse RNA species, including messenger RNA (mRNA), transfer RNA (tRNA), ribosomal RNA (rRNA), long non-coding RNA (lncRNA), microRNA (miRNA), and others (3, 4). RNA methylations are enzymatically regulated by specific methyltransferases (“writers”), demethylases (“erasers”), and recognition proteins (“readers” or “binders”), forming a sophisticated network analogous to epigenetic DNA regulation (5).

Autoimmune Rheumatic Diseases (ARDs) encompass a heterogeneous group of chronic inflammatory conditions, including rheumatoid arthritis (RA), systemic lupus erythematosus (SLE), ankylosing spondylitis (AS), primary Sjögren’s syndrome (pSS), and systemic sclerosis (SSc), characterized by aberrant immune activation targeting self-tissues, leading to inflammation, tissue damage, and significant morbidity (6).While the therapeutic targeting of DNA methylation has emerged as a prominent strategy in ARDs (7), RNA methylation itself has increasingly been recognized for its profound regulatory roles in fundamental cellular processes relevant to diverse diseases. Significantly, accumulating evidence reveals that dysregulation of RNA methylation pathways exerts profound influence on immune function and inflammation, processes central to specific RNA methylations, such as N^6^-methyladenosine (m^6^A), 5-methylcytosine (m^5^C), N^1^-methyladenosine (m^1^A), 7-methylguanosine (m^7^G), and N^6^,2’-O-dimethyladenosine (m^6^Am) have been implicated in ARDs pathogenesis. For instance, aberrant m^6^A modification has been linked to dysregulated T cell activation, altered cytokine production, and pathogenic autoantibody generation, hallmarks of ARDs (8, 9). Alterations in m^5^C and m^1^A modifications have also been associated with disease severity and outcomes in patients (10). However, the precise relationships between specific RNA modifications, metabolic pathways in immune cells, and the development or progression of ARDs remain incompletely understood. Given their dynamic nature and regulatory potency, RNA methylation pathways represent promising novel therapeutic targets for ARDs (11). Modulating “writer,” “eraser,” or “reader” activities offers the potential to correct aberrant gene expression profiles, suppress pathological inflammation, and restore immune tolerance. Furthermore, due to their stability and detectability in accessible biofluids, specific RNA methylation modifications hold significant promise as diagnostic and prognostic biomarkers for ARDs, enabling early detection, improved disease stratification, and monitoring of treatment response.

This review introduces the key internal RNA modifications and details their emerging roles in the pathogenesis of ARDs. We systematically discuss the current understanding of how dysregulation in RNA methylation contributes to ARDs like RA, SLE, AS, pSS, and SSc. We also explore the burgeoning therapeutic potential and diagnostic utility of targeting the RNA methylation machinery in these debilitating conditions.

## 2 Types of RNA methylation

To date, more than 170 distinct chemical modifications of RNA have been identified (12), with RNA methylation comprising approximately 60% of these (13). Unlike DNA methylation, a stable modification catalyzed by DNA methyltransferases (DNMTs) that primarily targets cytosines in CpG dinucleotides to repress transcription via heterochromatin formation, RNA methylation is a reversible post-transcriptional modification. It dynamically regulates RNA splicing, translation, stability, and degradation, thereby enabling fine-tuned control of immune-related gene expression (14). RNA methylation is a fundamental epigenetic mechanism involving the addition of methyl groups to RNA molecules, thereby modulating their structure, stability, and biological function. Among these modifications, m^6^A methylation at the nitrogen-6 position of adenosine remains the most extensively characterized. Other prevalent modifications include m^5^C, m¹A, m^7^G, m^6^Am, 5-hydroxymethyl-5-methylcytosine (hm^5^C), and m^3^C. m^6^A is the most abundant internal modification in mRNA, present in approximately 25% of mammalian cell mRNAs with an average of about three modification sites per transcript. In contrast, m^5^C is less abundant, occurring at levels three to ten times lower than those of m^6^A, at levels ranging from 0.03-0.1% of cytosines. m¹A is approximately ten times less abundant than m^6^A, detected at only a very limited number of sites with extremely low modification levels. m^7^G is even rarer, present at levels similar to those of m^1^A, that is, of ~0.04% of all guanosines. m^6^Am is primarily located at the 5′ cap structure of mRNA and is found at very low abundance as an internal modification. hm^5^C, an oxidation product of m^5^C, is present at very low levels, while evidence for the presence of 3-methylcytosine (m³C) in mRNA is limited, with this modification being more commonly observed in tRNA. In addition, Pseudouridine (Ψ), catalyzed by PUS enzymes, exhibits a Ψ/U ratio of approximately 0.2%–0.4% and is primarily located in coding sequences (CDS) and 3′UTRs (15, 16).

**Figure 1:**
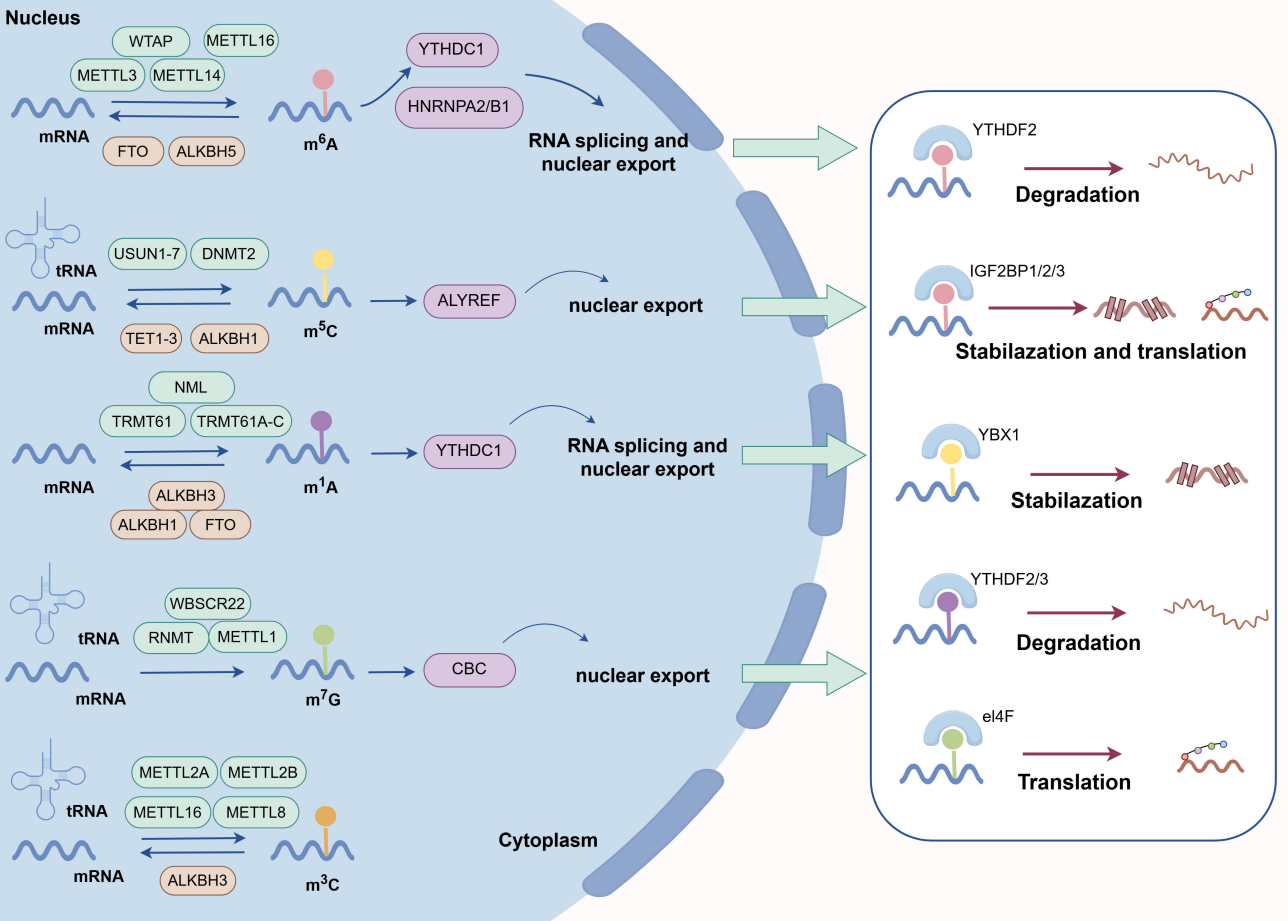
RNA methylation process. The important epigenetic modification, which affects RNA structure, stability and activity by adding methyl groups to RNA. Among them, m^6^A (at the N^6^ position of adenine) is the most studied modification. Other important modifications include m^5^C, m^1^A, m^7^G and m^3^C. These modifications are dynamically regulated by “writers” (methyltransferases), “erasers” (demethylases) and “readers” (binding proteins) (by Figdraw).

These chemical marks undergo dynamic regulation through methyltransferase “writers,” demethylase “erasers,” and binding protein “readers.” The modifications critically influence RNA processing mechanisms including splicing, translational control, degradation, and cell fate determination. Aberrant RNA methylation patterns correlate with pathological conditions such as ARDs (**Figure 1**).

### 2.1 m^6^A modification

m^6^A modification represents the most ubiquitous, abundant, and evolutionarily conserved internal cotranscriptional modification within eukaryotic RNA, with a particular prominence in higher eukaryotic cells (17). This modification occurs at the N^6^ position of adenosine, and remarkably, approximately one-third of the entire mRNA pool in mammals undergoes m^6^A modification (1, 2). Furthermore, m^6^A modification also occurs in rRNA (18), tRNA (19) as well as non-coding RNAs such as miRNA (20), lncRNA, small nuclear RNAs (snRNA) (21), and circular RNAs (circRNA) (22). Therefore, m^6^A modification influences RNA stability, splicing, translation, nuclear export, and other processes, playing a pivotal role in various biological functions and regulations of RNA.

The dynamic regulation of m^6^A primarily involves three core classes of enzymes: methyltransferases (writers), demethylases (erasers), and methylated RNA-binding proteins (readers). Methyltransferases responsible for adding m^6^A modifications to RNA molecules include METTL3, METTL14, WTAP, RBM15/15B, ZC3H13, VIRMA (KIAA1429), METTL16, METTL5, and ZCCHC4. METTL3 and METTL14 form a stable heterodimer that constitutes the core of the m^6^A methyltransferase complex. Within this complex, METTL3 primarily functions as the catalytic core, while METTL14 provides structural support, maintaining the heterodimer’s integrity and playing a pivotal role in recognizing RNA substrates (23). WTAP primarily interacts with the METTL3-METTL14 complex, facilitating its localization and enabling it to bind to optimal RNA substrates (24). Additionally, RBM15/15B (25), ZC3H13 (26), and VIRMA (27) serve as cofactors that, along with METTL3, METTL14, and WTAP, compose the complete methyltransferase complex, regulating m^6^A methylation and participating in the biological processes associated with RNA methylation. METTL16 functions specifically as the methyltransferase for U6 snRNA, playing a crucial role in RNA splicing. act as cofactors that, along with METTL3, METTL14, and WTAP, compose the methyltransferase complex to regulate m^6^A methylation, thereby participating in the biological processes of RNA methylation. METTL16 serves as the methyltransferase for U6 snRNA, playing a key role in RNA splicing (28). METTL5 and ZCCHC4 are essential rRNA methyltransferases. METTL5, which methylates 18S rRNA, requires binding with TRMT112 (a known methyltransferase activator) to form a heterodimer complex for stable existence and function within cells. Conversely, ZCCHC4 is responsible for the corresponding modification on 28S rRNA (29).

The demethylation of m^6^A is governed by demethylases like FTO and ALKBH5, enabling the reversal and regulation of this process. FTO is the first identified RNA demethylase capable of removing the methylation of m^6^A from mRNA both *in vitro* and *in vivo* (30). ALKBH5 represents the second RNA demethylase discovered to date, with m^6^A serving as its exclusive known substrate. It removes the m^6^A modification via an oxidative reaction, a process that exerts profound influences on mRNA export, metabolism, as well as the organization of mRNA processing factors within nuclear speckles (31).

The m^6^A methylation reader proteins constitute a diverse array of molecules, predominantly encompassing IGF2BP1/2/3 (32), YTHDF1/2/3 (33), ELAVL1 (34), and eIF3 (35). These reader proteins possess the ability to recognize and bind to RNA sites adorned with m^6^A modifications, subsequently initiating a cascade of downstream biological effects of RNA, including translation, splicing, nuclear export, and degradation. Furthermore, by modulating RNA stability and translation, they exert a profound influence on the initiation and progression of various diseases.

### 2.2 m^5^C modification

m^5^C is a pivotal RNA modification that primarily occurs in tRNA (36) and rRNA (37), yet its presence has also been documented in mRNA (38) and other non-coding RNAs (39, 40). This modification, characterized by the addition of a methyl group at the C5 position of cytosine, plays a crucial role in maintaining the stability, metabolism, nuclear export, and translation of the modified RNAs (41). m^5^C significantly impacts various biological processes, including cell proliferation (41), differentiation (42), migration (43), and apoptosis (44). Specifically, m^5^C has been demonstrated to be vital for tRNA stability, ensuring that tRNA molecules remain intact and functional throughout their lifecycle (45, 46). Furthermore, it enhances aminoacylation, a process by which tRNAs are attached to their corresponding amino acids, thereby facilitating accurate translation (47). Research has also illuminated the importance of m^5^C in maintaining translational fidelity, ensuring that proteins are synthesized with high accuracy (46). More recently, studies have hinted at a broader role for m^5^C in regulating mRNA export (48), translation (49) and stability (50), suggesting its involvement in a wide array of RNA metabolic processes.

The enzymatic addition of m^5^C is facilitated by “writers” such as DNA methyltransferase 2 (DNMT2) (51) and members of the NOP2/SUN RNA methyltransferase family, including NSUN1 to NSUN7 (52). Although DNMT2 is related to DNA methyltransferases, it is specific for RNA, particularly tRNA, catalyzing the formation of m^5^C within these molecules (53). Each member of the NSUN family plays a unique and critical role in RNA methylation, affecting cellular function and development, and being associated with diseases. NSUN1 is vital for the methylation of 28S rRNA at specific cytosine sites such as C4447 and plays a pivotal role in pre-implantation embryonic development by regulating nucleolar maturation and blastocyst formation (54). NSUN2, primarily located in the nucleus, methylates mRNA and various non-coding RNAs, adding m^5^C and influencing proliferation, stress response, metabolism, migration, and differentiation (44, 55–57). NSUN4 methylates 12S rRNA in mitochondria, facilitating the assembly of mitochondrial ribosomes (58). NSUN5 introduces m^5^C at the C3782 position of human 28S rRNA, impacting ribosome function, total protein synthesis, and cell proliferation (59). NSUN6 modifies specific motifs such as the CTCCA motif in the 3’ untranslated region of mRNA with m^5^C, increasing mRNA abundance and translation efficiency (60). Although less extensively studied, NSUN3 and NSUN7 are also known to catalyze the formation of m^5^C in various RNA species (61–63).

The “erasers” of m^5^C is responsible for removing the methyl group from RNA molecules, making RNA modification reversible. Currently known m^5^C demethylases primarily include TET1–3 and ALKBH1. TET1–3 belong to the TET enzyme family and function through an oxidative process, capable of oxidizing 5-methylcytosine (5mC) in RNA to produce 5-hydroxymethylcytosine (5hmC), which is a crucial step in RNA demethylation (63). On the other hand, ALKBH1 participates in the demethylation process of cytosolic tRNA, regulating the stability and translation efficiency of tRNA. The absence of ALKBH1 leads to a significant decrease in mitochondrial translation and oxygen consumption, highlighting its crucial role in regulating mitochondrial activity (64).

The “reader” proteins of m^5^C recognize and bind to RNA molecules containing this modification, directly influencing RNA function and fate. Currently known m^5^C reader proteins include YBX1, YBX2, FMRP, SRSF2, ALYREF, YTHDF2 and RAD52, among others. YBX1 and YBX2 are two proteins with RNA binding ability that can recognize and bind to mRNA molecules with m^5^C modifications. YBX1 plays a crucial role in maintaining the stability of its target mRNA marked by m^5^C (50, 65). FMRP, a fragile X mental retardation protein, is also an m^5^C reader protein. It can coordinate with m^5^C writers and erasers to promote mRNA-dependent repair and cell survival in cancer cells (66). SRSF2, a protein rich in serine/arginine and one of the core regulators of RNA splicing, has been shown to be a novel reader of m^5^C (67). In leukemia, common SRSF2 gene mutations (such as SRSF2P95H) impair its ability to read m^5^C -marked mRNA, thereby contributing to the onset of leukemia (67). Additionally, ALYREF (48), YTHDF2 (68), and RAD52 (69) are also m^5^C reader proteins that can recognize and bind to RNA molecules with m^5^C modifications. However, further research is needed to elucidate their specific roles in RNA metabolism.

### 2.3 m^1^A modification

m^1^A is an RNA modification characterized by the addition of a methyl group to the N^1^ position of adenosine. While less abundant than other modifications, m^1^A plays critical roles in RNA metabolism, particularly in tRNA (70) and rRNA (71). In tRNA, m^1^A is predominantly found at position 58 of the T-loop, a conserved structural motif essential for tRNA stability and proper folding (72). This modification enhances tRNA’s resistance to ribonucleases, ensuring its structural integrity and functional longevity (70). In rRNA, m^1^A modifications contribute to ribosome biogenesis and translational fidelity by stabilizing ribosomal subunits and optimizing rRNA-protein interactions (73).

The deposition of m^1^A is catalyzed by RNA methyltransferases (“writers”). The best-characterized m^1^A writer is the TRMT6/TRMT61A heterodimer, which specifically methylates adenosine at position 58 (A58) in the majority of cytoplasmic tRNAs (74). TRMT61A serves as the catalytic subunit, while TRMT6 stabilizes the complex and enhances substrate recognition (75). In mitochondria, the homologous enzyme TRMT61B introduces m^1^A modifications at A58 of mitochondrial tRNAs, ensuring their stability and functionality in mitochondrial translation (76). TRMT10C plays a pivotal role in catalyzing the methylation of a particular mA site in mitochondrial ND5 mRNA and participates in potential translational regulatory mechanisms through this modification (77). Additionally, NML is essential for the m^1^A modification of 28S rRNA in human and mouse cells and crucial for maintaining the structure and function of rRNA, which in turn affects the assembly and function of ribosomes.

The reversibility of m^1^A modification is mediated by demethylases (“erasers”). ALKBH1 and ALKBH3, members of the ALKB family of dioxygenases, are key m^1^A erasers. In mammals, ALKBH1 serves as a tRNA demethylase that specifically catalyzes the demethylation of m^1^A in tRNAs leads to a decrease in translation initiation and reduced utilization of the modified tRNAs in protein synthesis (78). ALKBH3 demethylates m^1^A in tRNA and mRNA, promoting tRNA degradation under stress conditions and enhancing mRNA translation by resolving methylation-induced structural constraints (79, 80). Additionally, FTO can directly inhibit translation by catalyzing the demethylation of m^1^A in tRNAs (81).

YTHDF1, YTHDF2, YTHDF3, and YTHDC1 have been identified as readers of m^1^A modifications, despite their primary roles as m^6^A readers (77, 82). Their binding affinity to m^1^A sites is weaker compared to that of m^6^A.

### 2.4 m^7^G modification

m^7^G is a prominent cap structure found at the 5’ end of eukaryotic mRNAs and select non-coding RNAs, such as stable nuclear RNA (snRNAs) and small nucleolar RNAs (snoRNAs). The m^7^G cap structure at the 5’ end of eukaryotic mRNA facilitates translation initiation, nuclear export of mRNA, and stabilization of mRNA structure (83, 84). The m^7^G modification also has significant impacts on the expression and function of non-coding RNAs (including tRNA (85), rRNA (86), and miRNA (87)), as it stabilizes the three-dimensional core structure of tRNA, aids in the processing of precursor miRNAs, and may play other roles that are not yet fully understood.

The deposition of m^7^G is mediated by distinct methyltransferases depending on RNA type. For mRNA capping, the canonical 5′ m^7^G cap is synthesized by a multi-step process involving RNA triphosphatase, guanylyltransferase, and methyltransferase activities. The RNMT-RAM complex catalyzes the methylation step in mammals, transferring a methyl group to the GpppN cap structure (88, 89). In tRNA, the METTL1-WDR4 heterodimer introduces m^7^G at position 46, a conserved site critical for tRNA folding and interaction with translation machinery (90, 91). For rRNA, the methyltransferase WBSCR22 (also known as BUD23), in complex with TRMT112, installs m^7^G modifications at specific sites in the 18S rRNA, facilitating ribosome assembly and export (92).

No direct m^7^G erasers have been definitively confirmed yet, so further research is required to elucidate the potential enzymatic reversal mechanisms.

Research on m^7^G reader proteins has made certain progress, with quaking (QKI) being the first identified m^7^G methyl-reading protein. Under stress conditions, QKI can recognize and bind to mRNAs with internal m^7^G modifications, and through interaction with G3BP1, it confines the target mRNAs within stress granules, thereby regulating their stability and translation efficiency (93).

### 2.5 m^6^Am modification

m^6^Am is a dual-methylation RNA modification involving methylation at both the N6 position of adenosine and the 2’-hydroxyl group of ribose. Predominantly found at the first transcribed nucleotide adjacent to the m^7^G cap (cap-proximal position) in mRNAs, m^6^Am also occurs internally in snRNAs and pri-miRNAs (94). This modification dynamically regulates RNA stability, translation, and processing, with emerging roles in stress adaptation and disease progression (95).

The deposition of m^6^Am is catalyzed by the methyltransferase PCIF1. PCIF1 specifically recognizes the m^7^G-capped RNA structure and methylates the adjacent adenosine at the N6 and 2’-O positions, generating the m^6^Am cap (96). For internal m^6^Am in snRNAs, the methyltransferase METTL4 has been implicated in depositing this modification, facilitating snRNA maturation and spliceosome assembly (97).

m^6^Am is reversibly regulated by the FTO, which selectively removes the N^6^-methyl group while leaving the 2’-O-methylation intact (98, 99).

Currently known m^6^Am methylation reader protein is PCF11. Researchers have identified PCF11 as an m^6^Am-specific reader in human cells using quantitative proteomics. m^6^Am functions by sequestering PCF11 away from RNA Pol II, thereby inhibiting PCF11 from dissociating RNA Pol II near transcription start sites and promoting full-length transcription of m^6^Am-modified RNAs (100).

### 2.6 hm^5^C modification

hm^5^C arises from the further modification of m5C in RNA. The TET enzyme family can convert m5C into hm^5^C (101, 102). hm^5^C plays a pivotal role in regulating lncRNA transcription (103). Despite its intriguing potential, the enzymes responsible for catalyzing hm^5^C formation and its precise functional roles in RNA metabolism remain largely unknown.

### 2.7 m^3^C modification

m^3^C is another RNA modification that, although less common than m^5^C, holds specific and important biological relevance. This modification takes place at the C3 position of cytosine and is catalyzed by specific RNA cytosine methyltransferases. The m^3^C modification plays a crucial role in maintaining the stability of tRNA structure (104).

m^3^C “writers” are a class of enzymes responsible for adding a methyl group to the third nitrogen atom of cytosine in RNA. In human cells, METTL2A, METTL2B, and METTL6 are primarily responsible for modifying m^3^C32 in cytoplasmic tRNAs. Among them, METTL2A and METTL2B share almost identical gene compositions and protein sequences, differing by only a few amino acids (105). Additionally, METTL6 can catalyze the formation of m^3^C at the C32 site of specific serine tRNA isoforms (106). Another important methyltransferase is METTL8, a mitochondrial protein that is responsible for methylating m^3^C at the C32 position of mitochondrial tRNAs, such as mt-tRNASer (UCN) and mt-tRNAThr (107). Finally, DALRD3 encodes a protein that forms a complex with METTL2 methyltransferase, which recognizes specific arginine tRNAs for m^3^C modification (104).

It has been confirmed that ALKBH3 can demethylate m^3^C in mRNA from mammalian cells *in vitro*, ALKBH3 significantly reduces the level of m^3^C in tRNA from HELA and 293T cells (80).

As for the reading proteins specific to m^3^C modification, detailed information is limited. With the advancement of research, more discoveries about m^3^C modification and its related enzymes and proteins are expected in the future.

## 3 Transcriptomic detection techniques for detecting RNA methylation

Transcriptome detection of RNA methylation employs sequencing methods analogous to those used in traditional genomic next-generation sequencing (NGS), characterized by shorter read lengths and faster processing speeds. Therefore, it is often referred to as an NGS-based detection technology (108). Methylated RNA immunoprecipitation sequencing (MeRIP-seq), a method based on RNA immunoprecipitation, uses specific antibodies and approximately 100 nt RNA fragments to detect m^6^A marks and can be flexibly applied to the analysis of other RNA modifications. Although antibody cross-reactivity may lead to false positives, MeRIP-seq is mainly used for the analysis of various methylation marks. However, this method has a lower resolution and the length of sequenced RNA fragments makes it difficult to precisely determine methylation sites, especially when there are multiple potential methylation motifs in a single fragment (109). Another method is m^6^A individual-nucleotide-resolution cross-linking and immunoprecipitation sequencing (miCLIP-seq), which utilizes ultraviolet cross-linking to achieve higher resolution and can distinguish m^6^A from m^6^Am (110). Another innovative approach is the evolved TadA-assisted N^6^-methyladenosine sequencing (eTAM-seq), which is an enzyme-assisted whole-genome sequencing technique that achieves base-level resolution through global deamination of adenosine and requires a very small sample size, only 10 cells (111). Additionally, for other types of methylation modifications in RNA, various detection methods have been developed, including RNA-BisSeq for m^5^C, m^7^G-seq, and m^1^A-quant-seq (112–114) (**Table 1**).

**Table 1:** Transcriptomic detection techniques for detecting RNA methylation.

Method	Feature	Ref.
MeRIP-seq	MeRIP-seq can achieve whole transcriptome sequencing with a relatively small sample size, but its resolution is relatively low.	(1)
miCLIP-seq	miCLIP uses UV-light cross-linking, with high resolution, and can distinguish between m^6^A and m^6^Am.	(110)
eTAM-seq	eTAM-seq, an enzyme-assisted sequencing technology that detects and quantifies m^6^A by global adenosine deamination.	(111)
RNA-BisSeq	This method achieves nucleotide-level resolution of m^5^C within the transcriptome range.	(112)
m^7^G-seq	m^7^G-seq achieved base resolution determination of m^7^G in mRNA and tRNA by converting internal m^7^G sites into abasic sites.	(113)
m^1^A-quant-seq	m^1^A-quant-seq achieved highly sensitive detection of m^1^A in low-abundance RNA by modifying HIV-1 reverse transcriptase.	(114)

## 4 The multifaceted roles of RNA methylation in ARDs

### 4.1 RNA methylation and RA

RA, a chronic inflammatory rheumatic disease primarily impacting joints, is marked by synovial hyperplasia, immune cell infiltration, and subsequent cartilage and bone destruction (115). Recent advancements in epigenetic research have illuminated the pivotal role of RNA methylation, particularly m^6^A in the pathogenesis of RA (116, 117).

The expression of m^6^A “writer” METTL3 and METTL14 is closely related to the pathogenesis and inflammatory response of RA (118–123). METTL3 is significantly increased in RA patients, and positively correlates with C-reactive protein (CRP) and erythrocyte sedimentation rate (ESR), two common biomarkers of RA disease activity. However, on the other hand, overexpression of *METTL3* markedly attenuates the LPS-induced inflammatory response in macrophages. METTL3 also facilitated the progression of RA via downregulating *RAC2* in an m^6^A dependent mechanism in TNF-α-treated MH7A cells. Mechanistically, METTL3 upregulated RAC2 via m^6^A methylation, and the METTL3/RAC2 axis activated the AKT pathway. Overexpressing RAC2 counteracted the inhibitory effects of METTL3 knockdown on cellular proliferation, motility, oxidative stress, and inflammation (124). These studies showed METTL3 exhibit both pro-inflammatory and anti-inflammatory effects in various cells and indicated that METTL3 may serve both as a promising biomarker for RA and as a novel therapeutic target for RA treatment. In the peripheral blood mononuclear cells (PBMCs) of patients with active RA, the levels of the METTL14 and m^6^A are decreased, and they correlate negatively with the disease activity score using 28 joint counts (DAS28) (120). One study suggested that METTL14 promotes FLSs activation and related inflammatory response via the LASP1/SRC/AKT signaling pathway (119). Knockdown of METTL14 results in decreased m^6^A levels and promotes the secretion of inflammatory cytokines including interleukin (IL)-6 and IL-17 in PBMCs of RA patients, with corresponding observations confirmed in the collagen-induced arthritis (CIA) mouse model (123). Overexpression of *WTAP* elevated WTAP and BCL2 expression while reducing BAX and TRAIL-DR4 levels, markedly suppressing MH7A cell apoptosis and enhancing viability and proliferation. Conversely, *WTAP* silencing produced opposing effects, indicating that WTAP regulates MH7A cell apoptosis through m^6^A-dependent modulation of TRAIL-DR4 (125). The expression of demethylases FTO and ALKBH5 can influence the progression of RA by affecting m^6^A methylation, which in turn impacts processes such as fibroblast-like synoviocyte (FLS) proliferation and T-cell immunity (126–131). Knockdown of *FTO* or treatment with an FTO inhibitor can inhibit the migration, invasion, and inflammatory response of RA FLS. Further research has confirmed that FTO promotes the stability of *ADAMTS15* mRNA in an m^6^A-IGF2BP1-dependent manner (131). The other study demonstrated reduced FTO expression in RA synovium, FLS, and CIA rat synovium and FLS. Overexpressing FTO suppressed RA progression by downregulating NSUN2 expression, increasing SFRP1 protein levels, and inhibiting the Wnt/β-catenin pathway (129). NLRP3 has been proven to be closely linked to the pathogenesis of RA, with significant expression observed in synovial tissues and FLS of RA patients. ALKBH5 can bind to NLRP3, and its silencing inhibits the proliferation of FLS and the levels of inflammatory cytokines. The mechanism involves inhibiting the mRNA level of *NLRP3* through m^6^A modification via the reader protein YTHDC2 (128). Additionally, the expression of ALKBH5 is increased in RA synovial tissues, CIA model rats, and RA FLSs, and a hypoxic environment enhances the expression of ALKBH5 in FLSs. Mechanistically, hypoxia-induced ALKBH5 expression promotes FLS aggression and inflammation by regulating CH25H mRNA stability (132). It is noteworthy that another study has revealed contrary findings, showing that the expression of demethylases FTO and ALKBH5 was significantly decreased in RA patients (133). The divergent outcomes likely arise from differences in the cellular microenvironment.

In addition, the roles of reading proteins in RA are gradually being discovered, such as IGF2BP1/2/3 (131, 134, 135) and YTH family proteins (136, 137). IGF2BP1/3 can participate in the inflammatory process of RA as a reader protein through m^6^A mediated by FTO and ALKBH5 (131, 138). IGF2BP3 can also promote RA progression by enhancing the expression of recombinant human ribonucleotide reductase small chain (RRM2) and regulating the migration and invasion of MH7A cells through the AKT/matrix metalloproteinase (MMP)-9 pathway (134). IGF2BP3 significantly influenced RA-FLS by enhancing cell proliferation, migration, invasion, and inflammatory cytokine secretion while suppressing autophagy. It also reduced ROS production through autophagy inhibition, attenuating macrophage inflammatory activation. Notably, RASGRF1-driven mTORC1 activation mediated IGF2BP3’s effects on both proliferation and inflammatory responses (139). YTH family proteins, functioning as reader proteins, participate in regulating the m^6^A methylation mediated by METTL3, FTO, and ALKBH5, thereby impacting biological processes such as inflammatory responses and FLS proliferation in RA (122, 128, 130). Furthermore, study revealed that YTHDC1, YTHDC2, and YTHDF2 are closely associated with the pathogenesis of RA. Among them, the expression level of *YTHDC1* negatively correlates with the number of CD8^+^ T cells and is significantly reduced in the synovial tissue of RA patients. Further functional experiments demonstrated that silencing *YTHDC1* in FLSs significantly inhibited their migration, invasion, proliferation, and induced apoptosis (137). YTHDC2 participates in METTL3-mediated m^6^A modification, thereby affecting AMIGO2 and enhancing the activation of RA-FLS (122). In addition, Lin et al. investigated the role of TGM2 in RA and assessed the potential of Sarsasapogenin (Sar) as a TGM2-targeted drug in FLS. They concluded that TGM2 is regulated by m^6^A methylation in RA-FLSs, and identified it as a potential target for fur RA treatment (140).

The expression of m^5^C methyltransferase NSUN2 is significantly increased in patients with RA and CIA rats. Knockdown of NSUN2 blocks the Wnt/β-catenin signaling pathway and inhibits RA pathological factors such as MMP3, fibronectin, and interleukins. Overexpression of the demethylase *FTO* inhibits RA by suppressing the expression of NSUN2, upregulating the level of SFRP1 protein, and blocking the Wnt/β-catenin signaling pathway (129).

A study integrated multiple technologies to systematically reveal the differential expression characteristics and immune regulatory mechanisms of m^6^A/m^1^A/m^5^C-related genes between RA and osteoarthritis (OA). Through differential expression analysis, 140 differentially expressed RNA modification-related genes were identified; combined with LASSO regression, RA-specific key genes (such as *ADAMDEC1*, *IGHM*) and OA-specific key genes (such as *OGN*, *TNFRSF11B*, *SCARA3*, *PTN*) were further selected. WGCNA linked RNA modification-related genes to pyroptosis-related modules, and PPI network analysis with the cytoHubba algorithm identified six hub genes (*CXCL10*, *CXCL9*, *CCR7*, *CCL5*, *CXCL1*, *CCR2*); the ceRNA network revealed their potential regulation by miRNA-lncRNA interactions. CIBERSORT analysis showed that in RA, M1 macrophages were strongly positively correlated with CXCL10 and CXCL9, while M2 macrophages were negatively correlated with CCR7, suggesting that these genes participate in RA pathogenesis by modulating immune cell infiltration. Single-cell RNA sequencing further localized the key genes to specific chondrocyte subpopulations (e.g., FC, preFC) and found that OGN, TNFRSF11B, and others were significantly upregulated in OA cells. Finally, IHC on synovial tissues from RA and OA patients and *in vivo* validation in a mouse DMM model confirmed that *ADAMDEC1* and *IGHM* were highly expressed in RA, whereas *OGN*, *TNFRSF11B*, *SCARA3*, and *PTN* were highly expressed in OA, providing protein-level validation of the bioinformatics screening results. This multi-omics integrative strategy not only revealed the differential immune regulatory networks and pyroptosis-related mechanisms involving RNA modification-related genes in RA and OA but also established a systematic analytical framework and experimental basis for identifying disease-specific diagnostic biomarkers based on m^6^A/m^1^A/m^5^C modifications (10).

**Figure 2:**
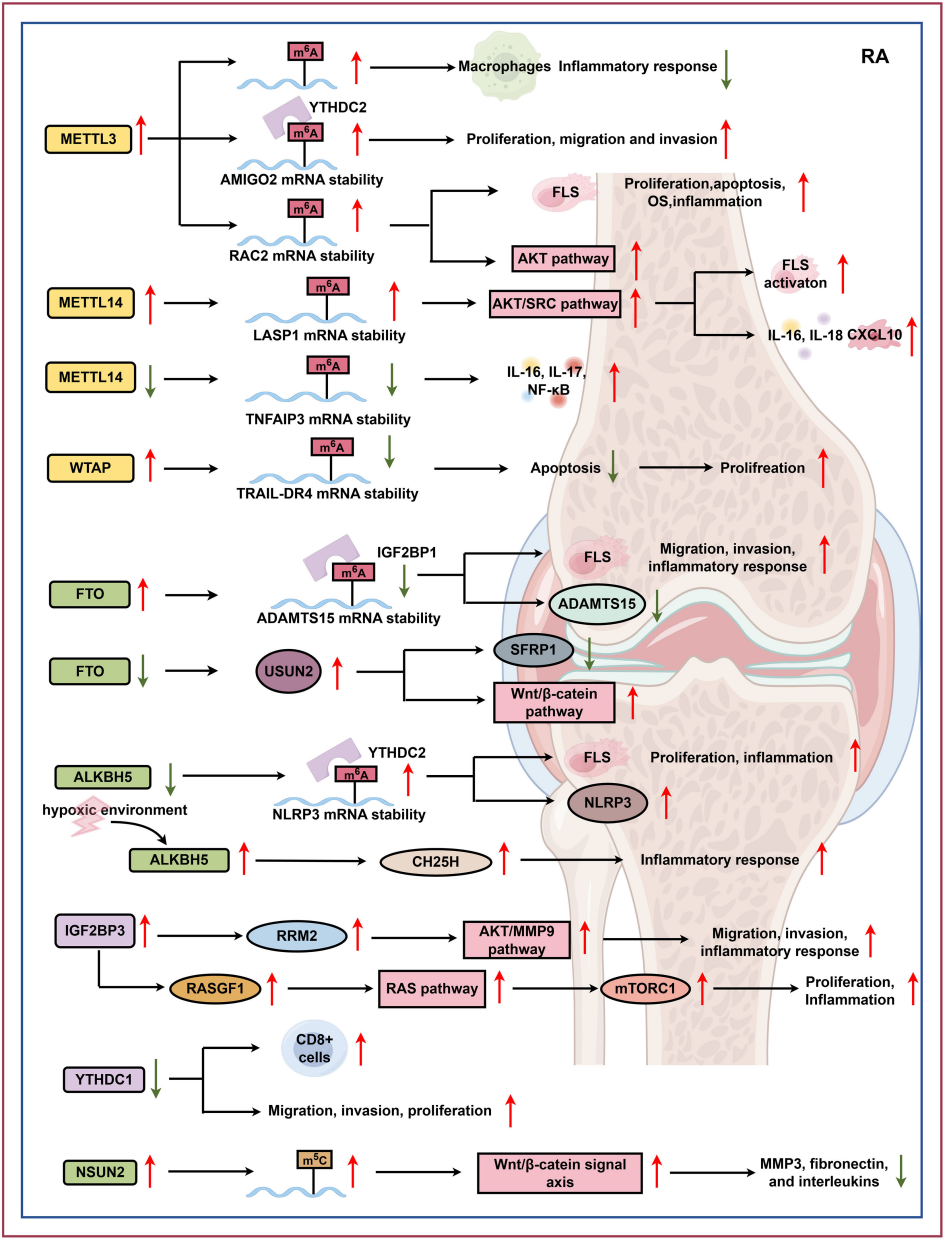
RNA modification in RA. RA, a chronic inflammatory disease primarily targeting the joints, is characterized by synovial hyperplasia, immune cell infiltration, and subsequent cartilage and bone degradation. Emerging evidence highlights the critical role of RNA modifications, specifically m^6^A, m^1^A, and m^5^C, in RA pathogenesis, suggesting their potential as diagnostic biomarkers. Dysregulation of the m^6^A modification pathway, including increased expression of writers (METTL3, METTL14, WTAP) and altered expression of erasers (FTO, ALKBH5) and readers (IGF2BP3, YTHDC1), is linked to RA progression. These alterations impact m^6^A methylation status, subsequently affecting key processes such as inflammation, fibroblast-like synoviocyte (FLS) proliferation, and T-cell immunity. Furthermore, increased expression of the m^5^C writer, NSUN2, is also associated with RA, with its knockdown inhibiting the Wnt/β-catenin signaling pathway and decreasing the production of RA-related factors like MMP3, fibronectin, and interleukins. Collectively, these key RNA modification regulatory factors (METTL3, METTL14, WTAP, FTO, ALKBH5, IGF2BP3, YTHDC1, NSUN2, etc.) influence mRNA stability and expression and control cellular processes, thereby representing potential therapeutic targets for RA intervention (by Figdraw). AMIGO2, adhesion molecule with Ig-like domain 2; RAC2, ras-related C3 botulinum toxin substrate 2; IL-16, interleukin-16; IL-17, interleukin-17; TRAIL-DR4, tumor necrosis factor-related apoptosis-inducing ligand death receptor 4; ADAMTS15, a disintegrin and metalloproteinase with thrombospondin motifs 15; SFRP1, secreted frizzled related protein 1; NLRP3, NLR family-pyrin domain containing protein 3; CH25H, recombinant cholesterol-25-hydroxylase; RRM2, ribonucleotide reductase M2; RASGF1, ras protein-specific guanine nucleotide-releasing factor 1; mTORC1, mechanistic target of rapamycin complex 1; MMP3, matrix metalloproteinase 3.

In summary, RNA methylation modulates gene expression, cytokine production, and immune cell activation through its regulation of inflammatory pathways. RNA methylation - particularly m^6^A modification - also directly impacts FLS proliferation, migration, and invasion. The altered expression of RNA-modifying enzymes such as METTL3, METTL14, FTO, ALKBH5, IGF2BP3, YTHDC1, YTHDC2, and NSUN2 correlates strongly with RA progression and disease severity (**Figure 2**). In addition, METTL3 has been reported to exhibit both pro-inflammatory and anti-inflammatory effects, while the expression trends of FTO and ALKBH5 are inconsistent across studies. These observations suggest that a single methyltransferase may have distinct, cell-specific functions.

### 4.2 RNA methylation and SLE

SLE is a typical rheumatic diseases that triggers autoantibody production, complement consumption, and chronic inflammation, impacting nearly every body part with a wide range of symptoms including skin manifestations, musculoskeletal symptoms, renal diseases, cardiac and pulmonary symptoms, as well as neuropsychiatric syndromes (141). The core pathogenic mechanism of SLE involves overactivation of interferon (IFN) -α signaling, production of anti-dsDNA antibodies, and neutrophil extracellular trap formation (NETosis). These factors collectively lead to uncontrolled immune attacks on the body’s own tissues, triggering widespread inflammatory responses and tissue damage. Dendritic cells, T helper cells, B cells, and plasma cells are all involved in this process (142). RNA methylation plays a pivotal role in immune response mechanisms (143), several studies have provided evidence of an association between RNA methylation and the pathogenesis of SLE.

In patients with SLE, the expression level of the m^6^A methyltransferase *METTL3* is significantly decreased in PBMCs and CD4^+^ T cells, leading to a reduction in overall m^6^A methylation levels. Further *in vitro* studies confirmed that inhibition of METTL3 reduces the stability of forkhead box protein p3 (foxp3) mRNA, thereby enhancing CD4^+^ T cell activation and suppressing the differentiation of regulatory T cells (Treg cells). This ultimately increases antibody production and exacerbates the lupus-like phenotype in cGVHD mice (144). Additional research demonstrates that METTL3 exacerbates SLE renal damage by catalyzing m^6^A modification of IRF4 mRNA, which increases its stability and expression level. This promotes plasma cell infiltration and antibody production (145). The demethylase ALKBH5 is closely associated with SLE. Studies show that ALKBH5 expression is significantly downregulated in PBMCs and T cells of SLE patients, and its expression level correlates closely with disease activity, complement levels, and autoantibody profiles. Specifically: In PBMCs, ALKBH5 mRNA levels show a positive correlation with plasma complement (C)-4 levels. In T cells, *ALKBH5* mRNA levels show a negative correlation with the SLE disease activity index (SLEDAI), ESR, and anti-dsDNA antibody titers, but a positive correlation with complement C3/C4 levels. Further functional experiments indicate that overexpression of *ALKBH5* significantly promotes T cell apoptosis and inhibits T cell proliferation, suggesting its potential role in SLE immune dysregulation through regulating T cell homeostasis (146). Another study shows that ALKBH5 catalyzes the m^6^A demethylation of forkhead box protein O1 (FoxO1) mRNA, which promotes FoxO1 binding to the promoter region of the Met gene, thereby suppressing Met gene transcription. This inhibition suppresses the Met/cyclooxygenase 2/prostaglandin E_2_ pathway, ameliorating B cell proliferation and hyperactivation in both SLE patients and lupus mouse models (147). The research conducted by Luo et al. indicates that the reduction of *METTL3*, *WTAP*, *ALKBH5*, *FTO* and *YTHDF2* may play a significant role in the pathogenesis of SLE. In addition, the expression of *ALKBH5* in peripheral blood may be involved in the pathogenesis of SLE, and it may also serve as a potential biomarker for the diagnosis and severity assessment of SLE (148, 149).

Furthermore, m^5^C methylation levels are significantly reduced in CD4^+^ T cells of SLE patients, accompanied by a marked decrease in expression of its methyltransferase NSUN2. Notably, the number of m^5^C-methylated mRNAs was unexpectedly increased. These methylated mRNAs are primarily enriched in key pathways related to the immune system and inflammation, such as cytokine signaling pathways and interferon signaling pathways (150).

To investigate the role of m^7^G in SLE immunopathology, Liu et al. examined the correlation between *METTL1* expression and clinical manifestations of the disease. Functional enrichment analysis of the GSE122459 dataset identified pathways associated with differentially expressed genes linked to *METTL1* expression. Single-sample GSEA further assessed the correlation between *METTL1* expression and immune cell infiltration to identify potential biomarkers. The results indicate that METTL1 and its candidate targets LAMP3, CD83, and PDCD1LG2 hold diagnostic value for SLE and could be explored for targeted molecular therapy development (151). In terms of mechanism, research has found that METTL1-mediated tRNA m^7^G modification controls abnormal B cell responses to autoantigens in SLE. Deficiency of *METTL1* alleviates dysregulated B cell responses during autoimmune induction (152).

**Figure 3:**
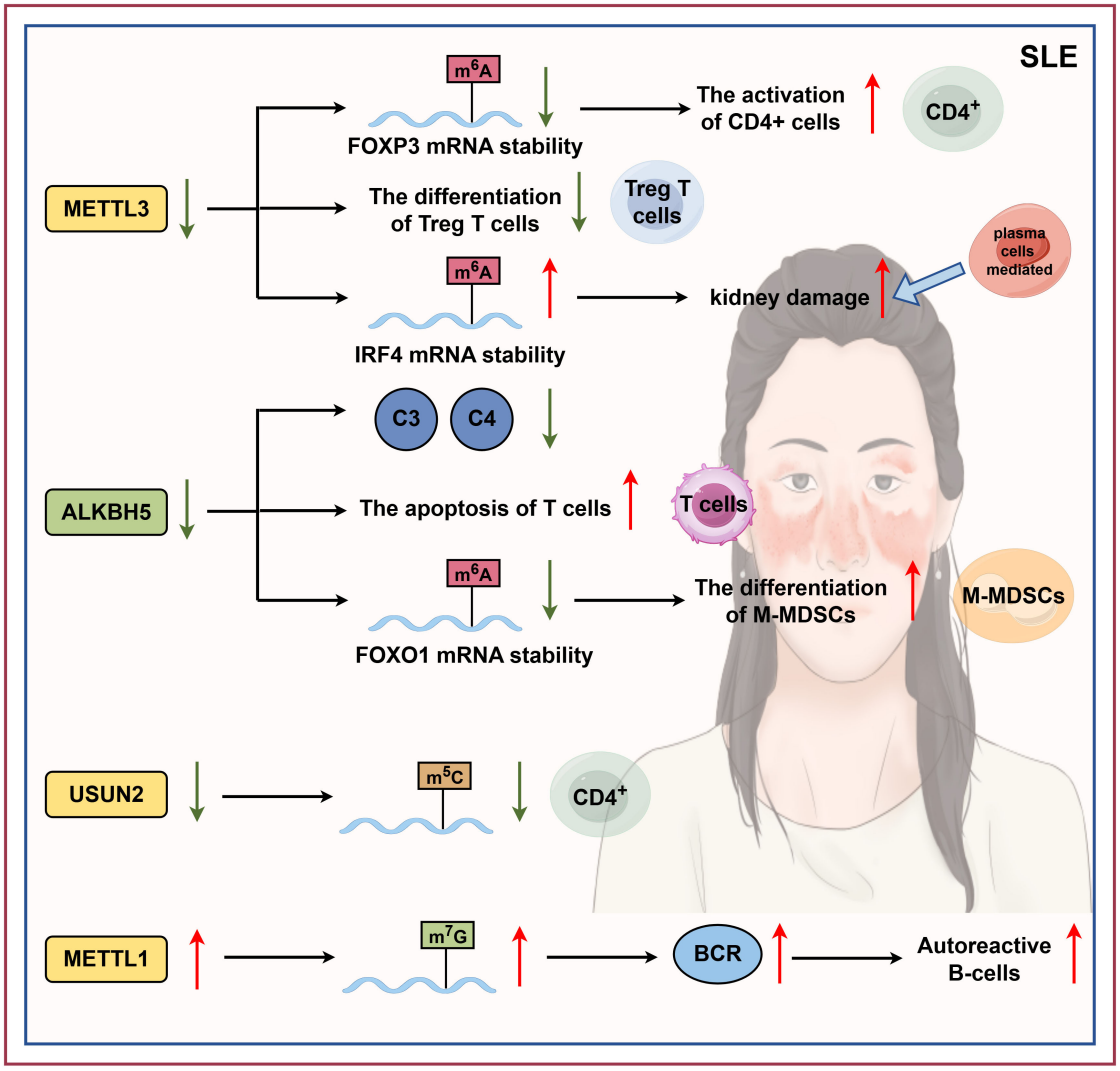
RNA modification in SLE. SLE is a systemic autoimmune disease defined by autoantibody production, complement activation, and chronic systemic inflammation. Emerging research suggests that RNA methylation alterations are central to SLE pathogenesis. Specifically, decreased levels of m^6^A methyltransferases (METTL3) and m^6^A demethylases (ALKBH5) are strongly linked to SLE. Moreover, m^5^C methylation levels are significantly reduced in CD4^+^ T cells from SLE patients. Finally, METTL1-mediated tRNA m^7^G modification is implicated in the abnormal B cell responses to autoantigens that characterize SLE (by Figdraw). FOXP3, forkhead box protein P3; IRF4, interferon regulatory factor 4; FOXO1, forkhead box protein O1; BCR, B cell receptor.

These findings establish RNA modification as a key regulatory mechanism in SLE pathogenesis, highlighting its influence on both innate and adaptive immune responses in disease progression (**Figure 3**).

### 4.3 RNA methylation and AS

AS develops relatively slowly, primarily characterized by syndesmophytes (bony outgrowths within ligaments) and spinal rigidity/ankylosis. Spinal ossification not only impacts normal bodily function but also significantly reduces quality of life (153). The pathogenesis of AS involves a complex interplay between HLA (specifically HLA-B27) and non-HLA genetic factors, including loci like endoplasmic reticulum aminopeptidase 1 (ERAP1) and IL-23 receptor (IL23R). Furthermore, gut dysbiosis, immune plasticity, and a variety of environmental factors (such as infections, heavy metals, stress, and cigarette smoking) are also implicated (154). These numerous non-genetic factors may exert a significant influence on epigenetic modifications.

**Figure 4:**
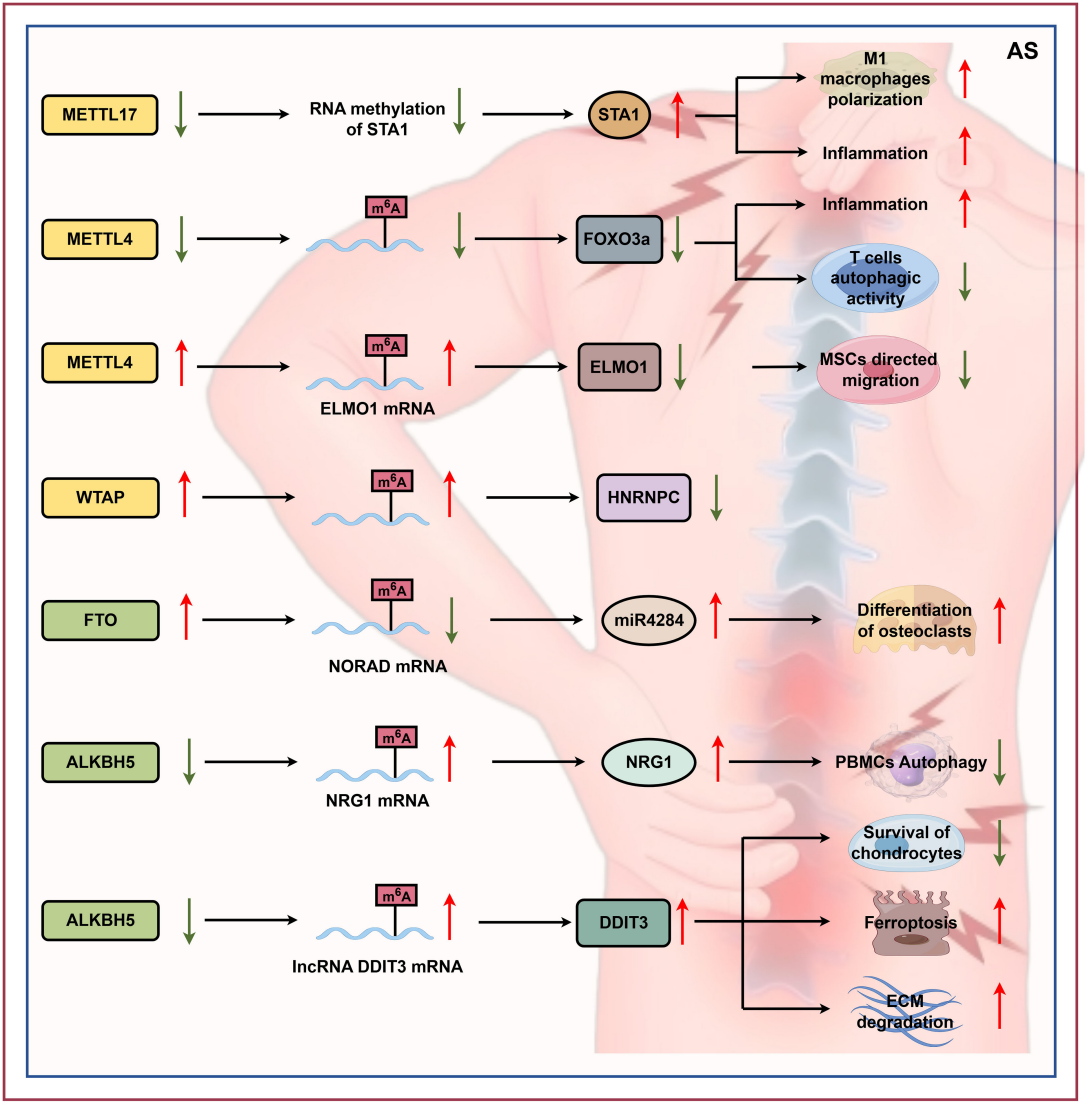
RNA modification role in AS. RNA modifications, particularly m^6^A methylation, play important roles in AS. METTL17 inhibits inflammation and M1 macrophage polarization by methylating STAT1 mRNA. Downregulation of METTL14 impairs autophagic flux via FOXO3a and exacerbates inflammation, while TNF-α-mediated m^6^A modification of ELMO1 regulates MSC-directed migration through METTL14 targeting of the ELMO1 3′UTR. The m^6^A methyltransferase WTAP is upregulated, whereas HNRNPC is downregulated in AS. The demethyltransferase FTO regulates AS-MSC function by controlling the m^6^A methylation state of the DNA-damage-activated lncRNA NORAD, thereby influencing its expression. Downregulation of ALKBH5 in AS increases m^6^A modification of lncRNA DDIT3 and NRG1 mRNA, enhancing their stability and expression, which promotes chondrocyte ferroptosis and suppresses PBMC autophagy, thus accelerating disease progression. Upregulation of YTHDF3 in AS enhances the stability of m^6^A-modified IL-32 mRNA in BMSCs, promoting their osteogenic differentiation and contributing to pathological bone formation (by Figdraw). STA1, heat-stable enterotoxin ST-IA/ST-P; FOXO3a, forkhead box protein O3a; ELMO1, engulfment and cell motility 1; HNRNPC, heterogeneous nuclear ribonucleoprotein C; NORAD, noncoding RNA activated by DNA damage; miR-4284, microRNA-4284; NRG1, neuregulin 1; DDIT3, DNA damage-inducible transcript 3.

The latest research indicates RNA methylation is involved in the pathogenesis of AS (**Figure 4**). According to Lv et al., METTL17 inhibits the inflammatory response and M1 macrophage polarization by mediating the RNA methylation of STAT1. Therefore, targeting METTL17/STAT1 may be a promising strategy for AS (155). Downregulation or deletion of METTL14 reduces m6A modification in T cells from AS patients, thereby decreasing Forkhead box O3a (FOXO3a) expression. This reduction impairs autophagic flux and exacerbates inflammation. The METTL14-m6A-FOXO3a axis thus represents a regulatory mechanism for autophagy and inflammation in AS, indicating its potential as a therapeutic target (156). Another study demonstrated that TNF-α upregulates ELMO1 expression in AS-derived mesenchymal stem cells (MSCs) via a METTL14-dependent m^6^A modification within the ELMO1 3’UTR. Consequently, suppressing *METTL14* elevates ELMO1 levels and enhances directional MSC migration, whereas *METTL14* overexpression reduces ELMO1 expression and impairs this migratory capacity (157). To further investigate the link between AS and m^6^A, Wu et al. performed methylation RNA immunoprecipitation sequencing (MeRIP-seq) and digital RNA sequencing (RNA-seq) on peripheral blood mononuclear cells (PBMCs) from three AS patients and three healthy controls. This analysis identified genes influenced by aberrant RNA methylation. The results indicate that *WTAP* is significantly upregulated in AS, whereas *HNRNPC* is markedly downregulated (158). Liu et al. discovered that the FTO was expressed more abundantly in MSCs of patients with AS compared to those from healthy donors. This study also revealed that FTO is a key regulatory factor that affects the function of AS-MSCs. Its mechanism of action lies in regulating the m^6^A methylation level of non-coding RNA activated by DNA damage (NORAD), thereby controlling the expression of NORAD. The research further found that silencing *NORAD* can enhance the formation of osteoclasts through miR-4284, suggesting that FTO may indirectly influence the generation of osteoclasts by regulating the methylation status of NORAD (159). Xie et al. found that in AS, decreased expression of ALKBH5 leads to increased m^6^A modification of lncRNA DDIT3, which enhances its stability and upregulates its expression. Elevated lncRNA DDIT3 acts as a molecular sponge for miR-142-3p.1, thereby modulating the HMGB1 axis. This cascade reduces chondrocyte viability, promotes ferroptosis, and exacerbates extracellular matrix (ECM) degradation, ultimately accelerating AS progression (160). Furthermore, another study showed that in AS patients, the m^6^A demethylase ALKBH5 is downregulated in PBMCs. This leads to increased m^6^A modification of NRG1 mRNA, which enhances its stability via the reader protein IGF2BP3, ultimately upregulating NRG1 expression. Elevated NRG1 levels suppress autophagy in PBMCs, thereby promoting AS progression (161). Zhou et al. demonstrated that in AS, YTHDF3 expression is upregulated in bone marrow mesenchymal stem cells (BMSCs). YTHDF3 binds to m^6^A-modified IL-32 mRNA and enhances its stability, leading to increased IL-32 expression. Elevated IL-32 promotes osteogenic differentiation of BMSCs, thereby contributing to pathological bone formation and AS progression (162).

### 4.4 RNA methylation and pSS

pSS is an autoimmune disease that causes systemic inflammation. In this disease, exocrine glands, such as the lacrimal glands and salivary glands, are affected, leading to patients experiencing dry mouth and dry eyes (163, 164). Studies have shown that activated salivary gland epithelial cells contribute significantly to the pathogenesis of pSS by initiating both immune and inflammatory responses. These cells facilitate immune cell infiltration while promoting autoantibody production and IFN-α expansion, ultimately leading to tissue damage (165).

**Figure 5:**
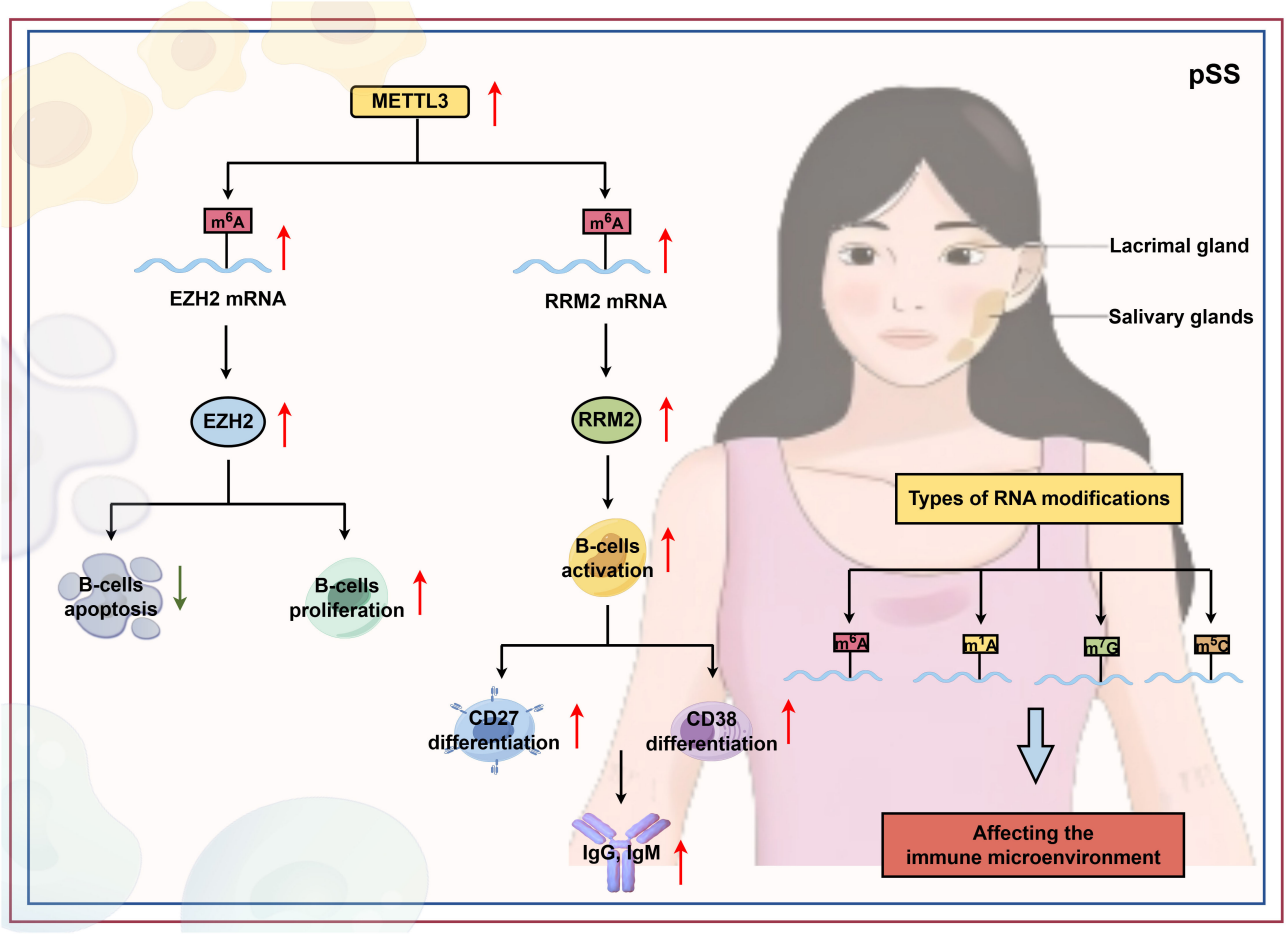
The role of RNA modification in the pathogenesis of pSS. pSS is an autoimmune disease marked by chronic systemic inflammation and the gradual destruction of exocrine glands, particularly the lacrimal and salivary glands. A central feature of the pSS immune microenvironment involves RNA modifications, including m^6^A, m^5^C, m^1^A, and m^7^G modifications, as well as their regulatory factors (by Figdraw). EZH2, ehancer of zste hmolog 2; RRM2, rbonucleoside-diphosphate reductase subunit M2.

As a widespread modification found in both coding RNA and non-coding RNA, m^6^A plays a crucial role in the functional regulation of immune cells (such as B cells and regulatory T cells), thereby influencing the progression of pSS to varying degrees (**Figure 5**). In a comparative study of 44 pSS patients, 50 healthy controls (HCs), and 11 non-SS sicca patients, the mRNA levels of m^6^A writers (*METTL3*, *RBM15*), erasers (*ALKBH5*, *FTO*), and readers (*YTHDF1-3*, *YTHDC1-2*) in PBMCs were significantly higher in pSS than in HCs. However, compared to non-SS sicca patients, pSS patients showed lower expression of *METTL3*, *WTAP*, *YTHDF2-3*, and *YTHDC2*. The expression of *METTL3*, *RBM15*, *FTO*, *YTHDF1-2*, and *YTHDC1–2* positively correlated with CRP, while *YTHDF1* negatively correlated with ESSDAI. *FTO*, *YTHDC1*, and *YTHDC2* were associated with white blood cell, neutrophil, lymphocyte, and monocyte counts. Elevated *ALKBH5* was identified as an independent risk factor for pSS. Moreover, *ISG15* expression positively correlated with *FTO*, *YTHDF2-3*, and *YTHDC2*, suggesting that these m^6^A regulators may activate type I interferon signaling and autoimmunity (166). Bioinformatics analyses have further elucidated the involvement of m6A modification in pSS. He et al. analyzed 23 m^6^A regulatory factors in parotid gland and blood samples, revealing significant expression differences between normal and pSS tissues. Cluster analysis identified distinct genetic and immune signatures associated with different m^6^A modification patterns, including differential immune cell infiltration. In parotid gland tissue, RNA metabolism and processing pathways were enriched, and KEGG analysis suggested a role for the autophagy pathway (167). Ma et al. reported that pSS patients with dry eye disease exhibited elevated m^6^A methylation levels and METTL3 expression in PBMCs compared to healthy controls (168). Multiple bioinformatics analyses confirmed strong correlations between m^6^A regulators (*ALKBH5*, *METTL3*, *RBMX*, *RBM15B*, *YTHDF1*) and immune cell infiltration/immune responses, indicating that m^6^A modification shapes the immune microenvironment in pSS (169, 170). Using machine learning, Yin et al. developed a predictive model incorporating three key m^6^A regulators (*METTL3*, *ALKBH5*, *YTHDF1*) and two m^6^A-related hub genes (*COMMD8*, *SRP9*), which accurately distinguished SS patients from controls (171). Additionally, Liu et al. demonstrated that m^6^A, along with m^5^C, m^1^A, and m^7^G modifications, forms a regulatory network that interacts with immune cells in the SS immune microenvironment (172).

Mechanistically, Yang et al. uncovered a critical METTL3-EZH2 axis driving B-cell autoimmunity in pSS. In pSS B cells, *METTL3* is upregulated and directly binds to *EZH2* mRNA, increasing its m^6^A modification and stability, thereby upregulating *EZH2* expression. Elevated EZH2 catalyzes H3K27me3 modification at the CDKN1A promoter, which suppresses B cell apoptosis and promotes B cell proliferation, ultimately exacerbating pSS disease activity (173). In a related study, Yang et al. showed that METTL3-mediated m^6^A modification enhances RRM2 mRNA stability, leading to increased RRM2 expression. Elevated RRM2 promotes B cell activation, facilitates differentiation into CD38^+^ CD27^+^ plasma cells, and augments the production of IgG, IgM, and antinuclear antibodies, further aggravating pSS (174).

### 4.5 RNA methylation and SSc

**Figure 6:**
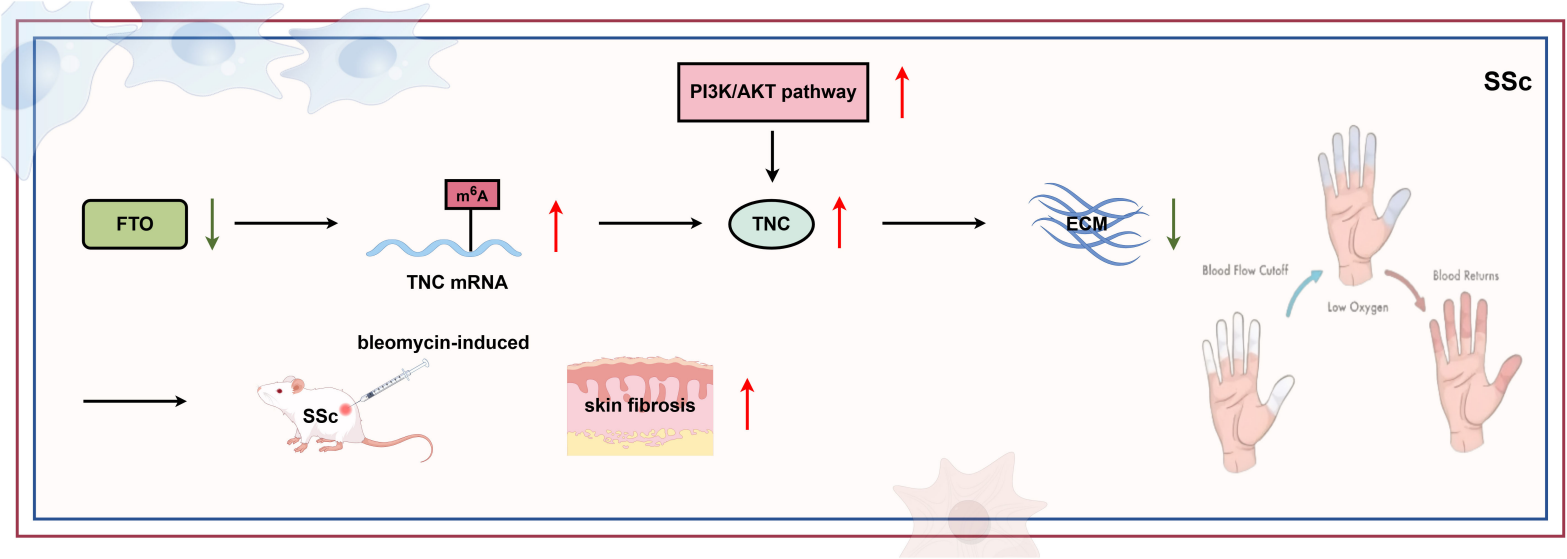
RNA modification in SSc. SSc, a progressive autoimmune disease characterized by widespread fibrosis in the skin and internal organs, is significantly influenced by RNA methylation. Specifically, m^6^A regulators play a crucial role in SSc development. FTO overexpression, for example, downregulates both the m^6^A and mRNA levels of TNC, thereby mitigating skin fibrosis (by Figdraw). TNC, tenascin-C.

SSc is a progressive autoimmune disease characterized by extensive fibrosis of the skin and internal organs. In the early stages of the disease, patients often exhibit abnormal accumulation of extracellular matrix in the skin of hands and feet, followed by fibrosis. As the disease progresses, fibrosis spreads upward to the forearms and legs; in the most severe diffuse SSc type, the skin of the trunk is also affected. What is even more noteworthy is that the pathological process of SSc goes beyond the skin; it also leads to tissue fibrosis in important internal organs such as the lungs, heart, and kidneys, severely affecting organ functions (175). The pathogenesis of SSc has not been fully elucidated yet. It is known that epigenetic factors play a crucial role in the ability of SSc myofibroblasts to maintain their phenotype during ex vivo tissue culture (176). Few studies have yet investigated RNA methylation in SSc. However, m^6^A regulators appear crucial in scleroderma pathogenesis (**Figure 6**). Investigations involving over 300 skin samples from scleroderma patients and studies on human dermal fibroblasts has demonstrated that overexpressing FTO can mitigate skin fibrosis by reducing the m^6^A modification and subsequent mRNA levels of tenascin-C (TNC). This highlights a potential avenue for therapeutic intervention in scleroderma (177). As SSc represents the most severe scleroderma subtype, these findings establish a foundation for exploring m^6^A modifications in SSc.

In summary, RNA methylation modifications play a significant role in the progression of ARDs. In different diseases, methyltransferases, demethylases, and reader proteins exert their respective functions by regulating distinct molecules, thereby modulating the biological functions of various cells. Detailed information on the mechanisms of action of RNA methylation in different ARDs is presented in **Table 2**.

**Table 2:** The role of RNA methylation in ARDs.

Types	Component	Diseases	Related targets	Biological function	Sample resources	Refs
m^6^A	METTL3↑	RA	CRP↑ ESR↑	Reduce inflammatory response.	THP-1 cells	(120)
m^6^A	METTL3↑ YTHDC2↑	RA	AMIGO2↑	Promote the proliferation, migration and invasion abilities of RA-FLS.	FLSs	(122)
m^6^A	METTL3↑	RA	RAC2↑	Promote oxidative stress and inflammatory responses in cells, and inhibit cell apoptosis.	MH7A cells	(124)
m^6^A	METTL14↑	RA	LASP1↑	Inhibiting cell apoptosis, promoting cell migration and invasion, and facilitating the production of IL-6, IL-18 and CXCL10 induced by TNF-α.	FLSs	(119)
m^6^A	METTL14↓	RA	TNFAIP3↓	Promote the secretion of IL-6 and IL-17 by PBMCs	PBMCs	(123)
m^6^A	WTAP↓	RA	TRAIL-DR4↑	Promote the apoptosis of MH7A cells, while inhibiting their survival and proliferation.	MH7A cells	(125)
m^6^A	FTO↑	RA	ADAMTS15↓	Promote the migration, invasion and inflammatory response of RA FLS.	FLSs	(131)
m^6^A	FTO↓	RA	USUN2↑ SFRP1↓	Inhibit the expression of NSUN2, increase the protein level of SFRP1, and block the Wnt/β-catenin signaling pathway.	FLSs	(129)
m^6^A	ALKBH5↓	RA	NLRP3↑	Inhibit the proliferation of FLS and the levels of inflammatory factors.	FLSs	(128)
m^6^A	ALKBH5↑	RA	CH25H↑	Promote the proliferation and migration of RA-FLS as well as the inflammatory response.	FLSs	(132)
m^6^A	IGF2BP3↑	RA	RRM2↑	Promote the expression of RRM2 and regulate the migration and invasion abilities of MH7A cells through the Akt/MMP-9 pathway.	MH7A cells	(134)
m^6^A	IGF2BP3↑	RA	RASGF1↑	Promote FLS proliferation, migration, invasion, release of inflammatory cytokines and inhibit autophagy.	FLSs	(139)
m^6^A	YTHDC1↓	RA	/	Inhibit the migration, invasion and proliferation of FLS, and induced cell apoptosis.	FLSs	(137)
m^6^A	METTL3↑	RA	TGM2↑	Activate the NF-κB signaling pathway, promote the proliferation of RA-FLS, and inhibit cell apoptosis.	FLSs	(140)
m^6^A	METTL3↑	RA	ICAM2↑	Activate the PI3K/AKT/p300 pathway, promoting the proliferation, migration, invasion of RA-FLS and inhibiting apoptosis.	FLSs	(178)
m^6^A	METTL3↓	SLE	Foxp3↓	Reduce Treg differentiation, promote the activation of CD4^+^T cells and the imbalance of effector T cells.	CD4^+^T cells	(144)
m^6^A	METTL3↑	SLE	IRF4↑	Promote the levels of serum creatinine, antinuclear antibody (ANA), and urinary albumin (ALB), intensify the deposition of IgG and C3, aggravate kidney damage, and cause plasma cell infiltration.	PBMCs	(145)
m^6^A	ALKBH5↓	SLE	C3/C4↓	Inhibit the apoptosis of T cells and promote their proliferation.	PBMCs	(146)
m^6^A	ALKBH5↓	SLE	FoxO1↓	Block the Met/ cyclooxygenase 2/prostaglandin E_2_ secretion pathway, promoting B cell proliferation and excessive activation.	MDSCs	(147)
m^5^C	USUN2	SLE	/	Regulation of translation and mRNA metabolism.	CD4^+^T cells	(150)
m^7^G	METTL1↓	SLE	LAMP3↓ CD83↓ PDCD1LG2↓	Related to immune cell infiltration, it has diagnostic value for SLE and can serve as a potential biomarker and therapeutic target.	/	(151)
RNA methylaton	METTL17↓	AS	STAT1↑	Promote the inflammatory response and the polarization of M1 type macrophages.	THP-1 cells	(155)
m^6^A	METTL14↓	AS	FOXO3a	Promote T cell inflammation and inhibit autophagic activity.	T cells	(156)
m^6^A	METTL14↑	AS	ELMO1↓	Inhibit the directional migration of MSCs.	MSCs	(157)
m^6^A	WTAP↑ HNRNPC↓	AS	BCL11B IL1R1	/	/	(158)
m^6^A	FTO↓	AS	NORAD↓	The reduction of FTO would disrupt the NORAD/miR-4284 axis, weaken the inhibition of MSCs on osteoclast formation, thereby losing bone protection and promoting bone destruction.	MSCs	(159)
m^6^A	ALKBH5↓	AS	lncRNA DDI3↓	Inhibit the survival of chondrocytes, promote ferroptosis, and facilitate the degradation of ECM.	Chondrocytes	(160)
m^6^A	ALKBH5↓	AS	NRG1↑	Inhibit autophagy of PBMCs.	PBMCs	(161)
m^6^A	YTHDF3↑	AS	IL-32↑	Promote the osteogenic differentiation of BMSCs.	PBMCs	(162)
m^6^A	METTL3↑	pSS	/	The expression of METTL3 is associated with anti-SSB antibodies, IgG, complement C3, and dry eye symptoms, suggesting that METTL3 may participate in the immune disorder and ocular damage of pSS dry eye through regulating m^6^A modification.	PBMCs	(168)
m^6^A	METTL3↑	pSS	EZH2↑	Inhibit B cells apoptosis and promote B cell proliferation.	B cells	(173)
m^6^A	METTL3↑	pSS	RRM2↑	Promote the activation of B cells, facilitate their differentiation into CD38^+^ CD27^+^ plasma cells, and increase the production of IgG, IgM and anti-nuclear antibodies.	B cells	(174)
m^6^A	FTO↓	SSc	TNC↑	Promote collagen deposition and aggravate skin fibrosis.	PBMCs, female Balb/c mice	(177)

Note: symbol ↑ means upregulation, and symbol ↓ means downregulation.

## 5 Targeting RNA modification systems for ARDs treatment

RNA modification systems have become highly attractive targets for drug intervention due to their reversible nature and cell-type specificity. In recent years, regulatory proteins centered on m^6^A modification (writers, erasers, and readers) have demonstrated therapeutic potential across various diseases. Notably, the small-molecule METTL3 inhibitor STC-15 has entered clinical trials, marking a crucial step in the translation of this field from foundational research to clinical application (179). This section systematically summarizes targeting strategies focused on m^6^A modification in ARDs and explores their potential applicability to other RNA modification types.

### 5.1 RA

Current m^6^A-targeting strategies for RA primarily focus on regulating demethylases; however, their effects are cell type-dependent, exerting therapeutic functions by either increasing or decreasing m^6^A modification levels.

In RA synovial fibroblasts, highly expressed FTO promotes disease progression by removing m^6^A modifications and reducing the stability of ADAMTS15 mRNA. The small-molecule inhibitor FB23–2 directly inhibits FTO activity, thereby increasing m^6^A modification levels and suppressing cell migration, invasion, and inflammatory responses, ultimately reducing arthritis severity in a CIA mouse model (131). Chen et al. used a CIA mouse model to confirm the anti-RA potential of artemisinin (ATT). The research results show that ATT inhibits RA progression by targeting the intercellular adhesion molecule 2 (ICAM2)/PI3K/AKT/p300 pathway in RA-FLSs. This effect involves ATT-mediated suppression of METTL3-dependent m^6^A methylation of ICAM2 mRNA within these cells. Furthermore, p300 positively regulates METTL3 transcription, a process that is also inhibited by ATT (178). METTL3, Thus, ICAM2 and p300 may serve as biomarkers for the treatment response of RA patients. The other study used MeRIP-seq and RNA sequencing (RNA-seq) based on the discovery of differentially methylated and expressed genes (DMEGs) in RA to analyze the potential targets of triptolide (TP). The results of molecular docking and *in vitro* experiments demonstrated a strong binding affinity between TP and IGF2BP3, and TP reduced IGF2BP3 mRNA expression in PBMCs and MH7A cells. These findings underscore the role of m^6^A modification in gene regulation in RA, suggesting IGF2BP3 as a potential therapeutic target and TP may is a potential therapeutic agent (180). These studies provide a reference for the development of small molecule drugs based on m^6^A research in RA.

These findings indicate that the m^6^A demethylases FTO and ALKBH5 serve as potential therapeutic targets in RA, but strategies involving inhibition or activation must be selected based on cell type and functional context.

### 5.2 SLE

In SLE, current research primarily focuses on targeting the methyltransferase METTL3. However, findings indicate that inhibiting METTL3 exacerbates disease progression, suggesting that METTL3 plays a protective role in this disease. Thus, activation rather than inhibition might represent a viable therapeutic approach.

In a SLE model, inhibition of METTL3 by STM2457 reduces m^6^A modification levels on Foxp3 mRNA in CD4^+^ T cells, accelerating its degradation and resulting in decreased Foxp3 expression, thereby impairing the differentiation of regulatory T cells (Tregs). *In vivo* studies show that STM2457 significantly promotes CD4^+^ T cell activation, reprograms effector T cell differentiation, and enhances autoantibody production, ultimately exacerbating lupus-like phenotypes in a chronic graft-versus-host disease (cGVHD) mouse model. These findings indicate that METTL3 exerts a protective role in SLE by using m^6^A modification to stabilize Foxp3 mRNA and maintain Treg differentiation. As a METTL3 inhibitor, STM2457 is unsuitable for SLE treatment and instead worsens disease progression (144). Therefore, there currently exists no genuine “m^6^A inhibitor” suitable for treating SLE; rather, activating METTL3 or preventing its downregulation may represent a viable therapeutic strategy.

RNA modification systems provide a novel landscape for therapeutic targeting, with the m^6^A pathway in particular demonstrating feasibility for translation from basic research to clinical application. Although numerous inhibitors or agonists targeting RNA methylation regulators have been developed, the majority are designed for oncology applications, including suppressing cancer cell proliferation and inhibiting oncogene translation (181–184). In contrast, there are relatively few inhibitors or agonists of RNA methylation regulators specifically developed for ARDs. This gap presents a promising direction for future drug development.

## 6 Future prospects for RNA methylation in ARDs

ARDs are a group of disorders caused by abnormal immune responses of the body to its own tissues or chronic inflammation. They mainly affect joints, muscles, and bones, and often involve damage to multiple organs such as the heart, lungs, and kidneys. The common types include RA, SLE, AS and SSc, etc. Usually, comprehensive treatment is required to control symptoms, slow down disease progression, and improve the prognosis of patients. Research on ARDs is now focusing on how to gain a deeper understanding of the molecular mechanisms of these complex diseases and develop more effective and personalized targeted treatments. Core challenges include improving the design of clinical trials, exploring and validating new biomarkers to achieve precision medicine, and developing innovative therapies for patients with refractory conditions (185).

RNA methylation has become a research hotspot in the field of epigenetics, and it is closely related to ARDs and may provide biomarkers and therapeutic targets for diseases (186). At present, RNA methylation research of ARDs mainly focuses on m^6^A and m^5^C, while studies on m^1^A, m^7^G, m^6^Am, hm^5^C, and m^3^C are relatively few. Moreover, current research mainly focuses on RA, SLE and AS, while RNA methylation studies on pSS and SSc are relatively scarce. In addition, the specific timing and manner by which RNA methylation regulatory proteins participate in the methylation process, their complex interactions with immune cells in different disease contexts, and whether there are synergistic or antagonistic effects between RNA methylation modifications, etc., remain key mechanisms that require further study. Therefore, a comprehensive understanding of these complex mechanisms requires systematic methods and tools for the rapid, accurate detection of RNA alterations. Innovative technologies are also needed to precisely manipulate transcriptomic epitopes and functions. This integrated approach will ultimately reveal the dynamic mechanisms governing different RNA modifications. Moving forward, systematic investigations into the effects of RNA modifications on the transcriptome and epigenome across diverse cell types of ARDs should be prioritized. Achieving this will require leveraging advanced high-throughput sequencing technologies (e.g., MeRIP-seq) to elucidate the molecular dynamics driven by RNA modifications. Recent advances in RNA detection—enabled by chemical and enzymatic strategies—also provide promising tools for unraveling the mechanisms of ARDs (187). However, significant challenges remain in RNA modification research. Current detection technologies are often limited by high costs, lack of a universally accurate method, and difficulties in real-time dynamic monitoring. Moreover, precise manipulation of RNA modifications—such as site-specific editing - faces persistent issues including off-target effects, low efficiency, and challenges *in vivo* delivery and functional validation. To overcome these barriers, future efforts should focus on: (1) Detection technologies: Developing multimodal platforms integrating spatiotemporal transcriptomics and single-molecule real-time detection for rapid, high-throughput, and spatially resolved RNA modification mapping. (2) Precision tools: Engineering advanced RNA editors (e.g., optimized Cas13 systems, RNA base editors), programmable RNA-binding proteins (RBPs), and dynamic probes (e.g., live-cell imaging, optogenetic tools) to enable accurate, efficient, and tunable RNA manipulation. (3) Delivery & analysis: Designing safe and efficient *in vivo* delivery systems, coupled with computational biology and AI-driven approaches to enhance prediction, design, and integrative analysis of RNA modification dynamics.

In ARDs, RNA methylation shows promise as a diagnostic biomarker, but its direct mechanistic links to disease pathogenesis remain poorly understood. Future studies should prioritize: (1) Quantitative profiling of RNA methylation dynamics and their correlation with established pathological pathways to establish causality. (2) Elucidating the precise role of RNA methylation in ARD progression. In-depth investigation of key regulatory factors and hub genes may yield novel biomarkers for early diagnosis, precision therapy, and prognosis assessment of ARDs and their complications. (3) Exploring RNA methylation in immune cells and its immunomodulatory effects could unlock innovative therapeutic and diagnostic strategies.

In recent years, various small-molecule inhibitors targeting writers, erasers, and readers have been successfully developed, some of which have advanced to clinical research stages. Few clinical trials have yet explored RNA modification therapies for ARDs such as RA and SLE, highlighting the need to translate basic research findings into clinical applications. In addition to ARDs, m^6^A regulatory proteins have demonstrated broad therapeutic potential in fields such as oncology, immunotherapy, and stem cell therapy. Therefore, RNA methylation-based immunotherapies for ARDs represent a promising therapeutic direction. Elucidating key regulators and hub genes could yield precision medicine targets while advancing the development of targeted drugs to improve clinical outcomes.
